# Adeno-associated Virus Virus-like Particle Characterization via Orthogonal Methods: Nanoelectrospray Differential Mobility Analysis, Asymmetric Flow Field-Flow Fractionation, and Atomic Force Microscopy

**DOI:** 10.1021/acsomega.1c01443

**Published:** 2021-06-15

**Authors:** Samuele Zoratto, Victor U. Weiss, Gernot Friedbacher, Carsten Buengener, Robert Pletzenauer, Alexandra Foettinger-Vacha, Michael Graninger, Guenter Allmaier

**Affiliations:** † Institute of Chemical Technologies and Analytics, TU Wien (Vienna University of Technology), Vienna A-1060, Austria; ‡ Pharmaceutical Sciences, Baxalta Innovations (part of Takeda), Vienna A-1221, Austria

## Abstract

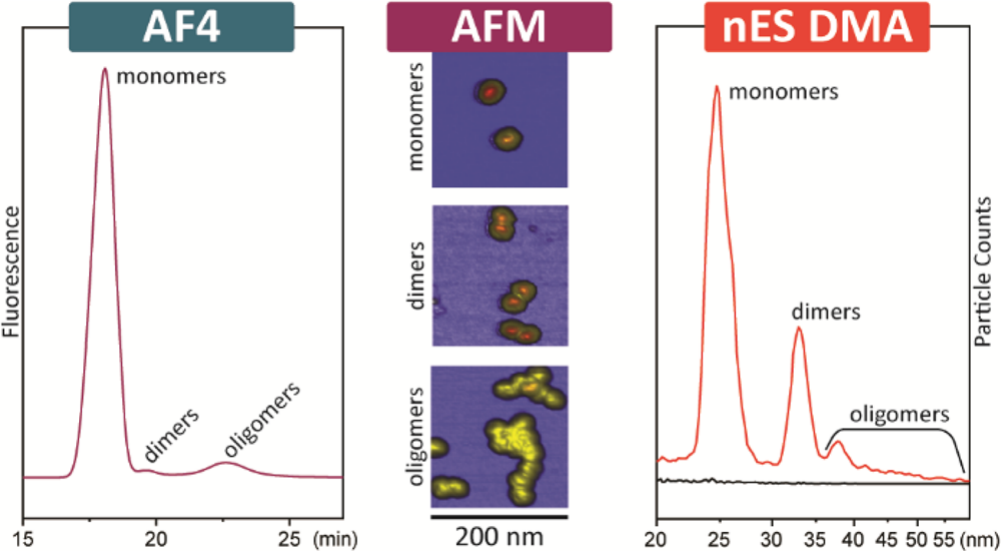

Adeno-associated virus (AAV)-based virus-like particles (VLPs) are thriving vectors of choice in the biopharmaceutical field of gene therapy. Here, a method to investigate purified AAV serotype 8 (AAV8) batches via a nanoelectrospray gas-phase mobility molecular analyzer (nES GEMMA), also known as an nES differential mobility analyzer, is presented. Indeed, due to AAV’s double-digit nanometer scale, nES GEMMA is an excellently suited technique to determine the surface-dry particle size termed electrophoretic mobility diameter of such VLPs in their native state at atmospheric pressure and with particle-number-based detection. Moreover, asymmetric flow field-flow fractionation (AF4, also known as AFFFF) and atomic force microscopy (AFM) techniques were employed as orthogonal techniques for VLP characterization. In addition, AF4 was implemented to size-separate as well as to enrich and collect fractions of AAV8 VLPs after inducing analyte aggregation in the liquid phase. Bionanoparticle aggregation was achieved by a combination of heat and shear stress. These fractions were later analyzed with nES GEMMA (in the gas phase) and AFM (on a solid surface). Both techniques confirm the presence of dimers, trimers, and putative VLP oligomers. Last, AFM reveals even larger AAV8 VLP aggregates, which were not detectable by nES GEMMA because their heterogeneity combined with low abundance was below the limit of detection of the instrument. Hence, the combination of the employed orthogonal sizing methods with the separation technique AF4 allow a comprehensive characterization of AAV8 VLPs applied as vectors.

## Introduction

In the biopharmaceutical field of gene therapy, one of the most investigated carriers is represented by adeno-associated virus (AAV) virus-like particles (VLPs)^1^ owing to their low immunogenicity, high efficiency of transduction, and transgene persistence in a broad range of tissues for *in vivo* applications.^2,3^ AAV is a helper-dependent virus of the *Parvoviridae* family, formed by 12 serotypes that show different tissue-specific tropisms.^4^ AAV is based on a non-enveloped, icosahedral capsid with a diameter of approximately 25 nm as related by cryoelectron microscopy reconstruction.^5^ Its cargo capacity is reported to be 4.7 kb of single-stranded DNA (ssDNA).^6,7^ The studies presented here were performed with purified (i.e., VLPs that are homogeneous in size, stable, and lacking aggregates) AAV serotype 8 (AAV8) either lacking (i.e., empty, which means a classical VLP) or carrying engineered ssDNA (i.e., filled particles).

The application of a nanoelectrospray gas-phase electrophoretic mobility molecular analyzer (nES GEMMA aka nES DMA (differential mobility analyzer), MacroIMS (ion mobility spectrometer), and LiquiScan ES or ES SMPS (scanning mobility particle sizer)) for size characterization of globular proteins has already been demonstrated.^8^ Furthermore, the applicability of this technique for the analysis of viral samples and their complexes (i.e., virus–antibody complexes) has already been confirmed in several studies.^9−14^ As its name suggests, a native nanoelectrospray process is involved in sample droplet formation. Dry analyte separation occurs by means of a differential mobility analyzer (DMA) in the gas phase, and particle detection is conducted using an ultrafine condensation particle counter (CPC) device. In detail, a cone-tipped fused silica capillary, tapered by a homebuilt grinding machine,^15^ is used to electrospray a volatile, aqueous electrolyte solution in which the viral analytes are dissolved or suspended. The liquid of the generated nanodroplets evaporates in a mixture of dry particle-free air and carbon dioxide. Simultaneously, charge equilibration is obtained by a bipolar atmosphere produced by, e.g., a radioactive source (i.e., ^201^Po, an α-particle emitter).^16^ Thus, a so-called “polydisperse aerosol” composed of surface-dry, single charged bionanoparticles is generated. This polydisperse aerosol is subsequently fed to the nano-DMA via the same air–CO_2_ mixture. Here, a well-defined, orthogonal electric field with increasing/decreasing voltage is applied in conjunction with a constant, particle-free, high laminar sheath air flow. Hence, the particles are sorted according to their different electrophoretic mobility diameters (EMDs), the applied voltage, the flow rate, and the DMA’s geometry. Therefore, for a given voltage and a fixed flow rate, a so-called “monodisperse aerosol” is produced, which is composed of nanoparticles with the same EMD. In the case of spherical particles, the EMD corresponds to the nanoparticle diameter. Once in the CPC device, the size-separated nanoparticles act as condensation nuclei in the supersaturated atmosphere of either water or *n*-butanol. Thus, droplet formation occurs. Subsequently, droplets are detected via light scattering optics.^17^ When a droplet crosses the focused laser beam, independently from its chemical composition or its original size, a count/signal is added to the spectrum at the relative EMD.^18^ Therefore, for a defined flow rate in the DMA, a specific range of EMDs can be explored. By variation of the applied electrical field, a GEMMA spectrum is generated,^19^ yielding number-based particle concentrations in accordance with a recommendation of the European Commission for nanoparticle characterization (2011/696/EU from October 18th, 2011). Previous works from Weiss et al.,^20^ Bereszczak et al.,^21^ Havlik et al.,^22^ and Pease et al.^23^ show that nES GEMMA is a suitable instrumentation for VLP analysis, and in this study, it has been implemented to develop a method to characterize purified AAV8 VLPs either with or without induced bionanoparticle aggregation.

Asymmetric flow field-flow fractionation (AF4, also known as AFFFF) is a soft liquid-phase separation technique that retains the native structure and conformation of the analytes. A detailed overview of the technique and its theory can be found elsewhere.^24,25^ In essence, sample separation is achieved on the intrinsic size-dependent diffusion coefficient of the analytes against an orthogonal crossflow force, and hence the separation is a function of the hydrodynamic radius of the sample components. Its implementation for the separation of bionanoparticles such as viruses and virus-like particles has already been verified in several studies.^23,26−31^ In this study, AF4 was employed to separate and collect the fractions of empty AAV8 VLPs at different hydrodynamic diameters representing monomeric, dimeric, and oligomeric states (formed after treating the original sample with heat and shear stress).

Furthermore, images of both empty and filled AAV8 VLPs samples were produced by means of atomic force microscopy (AFM). This device is capable of measuring in the subnanometer range; hence, it can easily produce images at the viral scale.^32−34^

A detailed description of the technique is reported elsewhere.^32^ In principle, a cantilever with an atomically sharp tip interacts with the sample surface. The local forces between the tip and the structural features of the sample displace the tip vertically. This movement is detected by the instrument and processed to generate an image. In this study, the instrument was set in tapping mode to acquire AFM images due to the softness of the nanoparticles. Thus, the tip is not in continuous contact with the sample surface, but instead, it gently oscillates rapidly on the surface. This approach is particularly indicated for samples that are too soft or too fragile for the continuous contact mode.^33^

**Figure 1:**
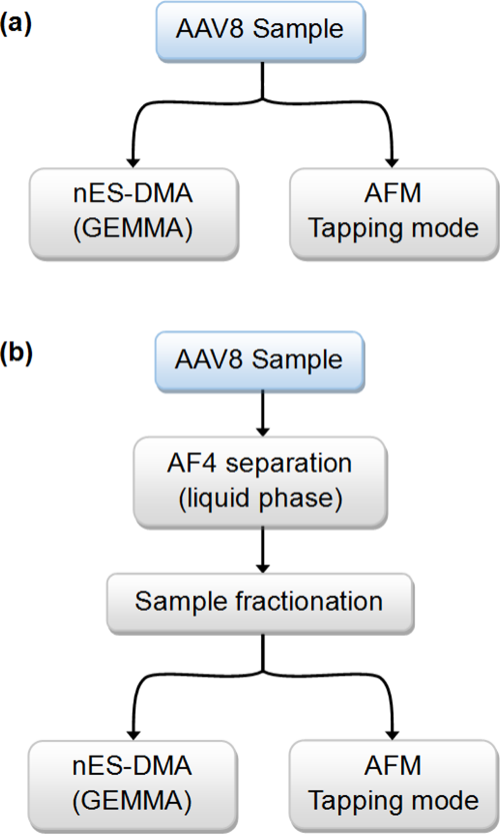
Scheme of the two analytical focuses of this study. (a) Empty and filled AAV8 VLP particles are analyzed via nES GEMMA and AFM. (b) AF4 liquid-phase separation enables AAV8 VLP oligomer analysis by means of nES GEMMA and AFM after fractionation.

The implementation of the described analytical techniques to investigate the size characteristics and the aggregation behavior of purified AAV8 VLPs is presented here with two strategies, as outlined in Figure 1. The first approach (see Figure 1a) is designed for the investigation of the differences between the two VLP preparations. This approach involves the usage of nES GEMMA and AFM techniques. The second approach (see Figure 1b) focuses mainly on the analysis of empty AAV8 VLPs and their aggregation behavior after bionanoparticle stressing by means of heat and mechanical agitation. With this method, AF4 was used to fractionate and collect the VLPs in different oligomeric states, which were later analyzed via nES GEMMA and AFM. Separately, nES GEMMA and AF4 were performed to prove why a buffer exchange step prior to sample analysis is crucial, even if it comes at the expense of bionanoparticle loss in this process.

## Results and Discussion

Our article aims to demonstrate the strategy for the analysis of an AAV-based VLP by means of nES GEMMA, AF4 with fluorescence detection, and AFM in tapping mode. Two strategies, summarized in Figure 1, have been tested. The first includes the analysis of both empty and filled AAV8 VLPs by means of nES GEMMA and AFM (see Figure 1a). The aim is to investigate and highlight the differences between the two preparations. Whereas, the second strategy (see Figure 1b) aims to investigate different oligomeric states of empty AAV8 VLPs generated under stress conditions. These oligomeric states are separated via AF4. The resulting fractions (representing monomeric, dimeric, and oligomeric bionanoparticles) are collected and later analyzed by nES GEMMA and AFM. Moreover, we also demonstrate the necessity of a volatile electrolyte solution for nES GEMMA. Therefore, frequently, a buffer exchange step is necessary. Hence, we also investigated the sample recovery of this procedure via AF4.

## Buffer Exchange and Sample Recovery

Nanoelectrospray gas-phase mobility molecular analyses are based on an electrospray process to transfer analytes from the liquid to the gas phase at atmospheric pressure. Hence, all nonvolatile sample components, e.g., from employed buffer solutions, affect results by generating additional peaks or by forming nonspecific interactions with the intended analyte, i.e., AAV8 VLPs. In the worst case, in terms of gas-phase electrophoresis, other nonvolatile sample components are in a much higher concentration than the analyte in question. Therefore, during the electrospray process, these components are forming nonspecific aggregates, influencing the baseline and complicating the spectra.^35^ This effect will be further elaborated in the following paragraph.

**Figure 2:**
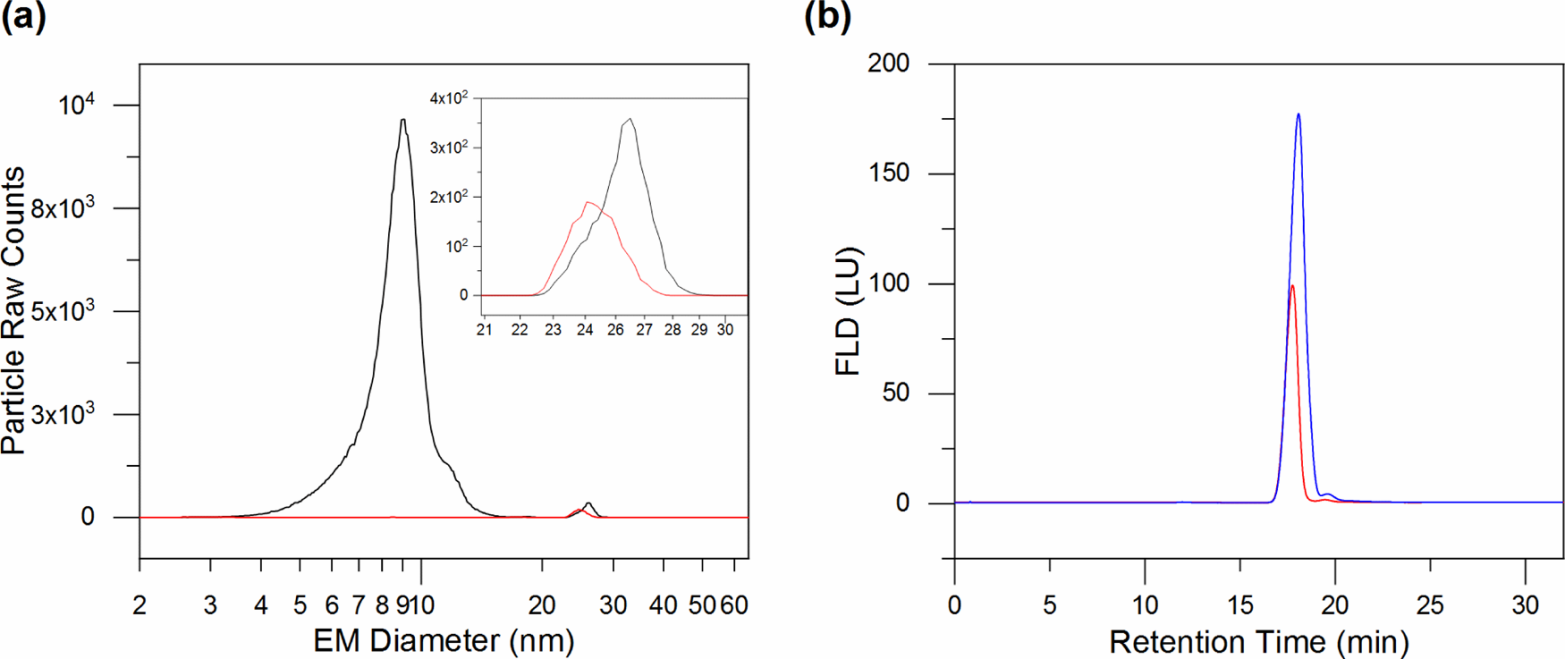
Implications of the buffer exchange treatment. (a) nES GEMMA spectra of the sample before (black trace) and after (red trace) desalting. Nonvolatile salts and other components (3–15 nm) are drastically reduced. The inset shows the shift toward small EM diameter of the AAV8 VLP’s peak. (b) AF4 fractogram of the sample with (red trace) or without (blue trace) a buffer exchange procedure. Both nES GEMMA spectra (a) and (b) show reduced signal intensity for the AAV8 VLP’s peak due to sample loss during the desalting treatment.

The effects of nonvolatile sample components other than the analyte are shown in Figure 2a, where the black profile shows the nES GEMMA signal produced after a 1:100 [v/v] dilution from the stock solution of empty AAV8 VPLs in NH_4_OAc. The dominant peak at approximately 9 nm EM diameter is mainly composed of all the components of the original buffer solution and probably in minor part by capsid’s fragments generated by degradation of the sample over time and handling of the stock solution. Moreover, nonspecific interactions between the buffer’s components and the sample are noticeable by the EM diameter difference between the two profiles of the peak at 25 nm (see the inset). This peak correlates to the AAV8’s capsid surface-dry particle diameter. For the VLP sample after simple dilution, an EM diameter of 25.94 nm with an SD of ±0.03 nm based on the particle number distribution is obtained. In contrast, when the VLP sample is subjected to buffer exchange (red trace), the EM diameter is reduced by about 1 nm (see data below). This behavior can be explained by the presence of salts and other nonvolatile components that unspecifically attach to the capsid. Hence, the buffer exchange step is a necessary prerequisite for reliable nES GEMMA. Indeed, our buffer exchange procedure removes efficiently original buffer components or other contaminants and retains AAV8 VLPs as dominating nanoparticle species. Nonetheless, lower signal intensity is evident for AAV8 VLP samples after buffer exchange, despite being allegedly diluted to the same concentration. This behavior is a consequence of the buffer exchange step itself. During this procedure, the capsids may unspecifically interact with the centrifugal filter membrane. Alternatively, by depleting the long-term stabilizing components of the original buffer, the capsids may adsorb on the vials’ inner surface or form nonspecific ultralarge aggregates. Most likely, the sample’s recovery is affected by all of these interactions, which in the end reflects a lower number of VLPs detected.

AF4 analyses further demonstrate that the lower AAV8 peak intensity is indeed due to incomplete analyte recovery during the buffer exchange step. As shown in Figure 2b, the fractrogram of the desalted sample (red profile) produces a lower fluorescent signal when compared with the fractrogram of the diluted sample (from the original stock, blue profile), despite the fact that for both samples, the same amount of analyte was injected (approximately 10 μg, based on an assumed total recovery of VLPs from the filter membrane after desalting). However, in contrast to nES GEMMA, buffer exchange is not necessary for AF4 since the sample’s buffer components do not interfere with the method of separation. On the other hand, no direct size information can instantly be gathered from AF4 analysis. The results from both GEMMA and AF4 techniques allowed us to estimate that the implementation of the buffer exchange step, although critical for nES GEMMA, causes a loss of 40% of the original AAV8 VLP content. The sample composition in terms of VLP monomers and oligomers remains constant (see Figure S1).

## Empty and Filled AAV8 VLPs

**Figure 3:**
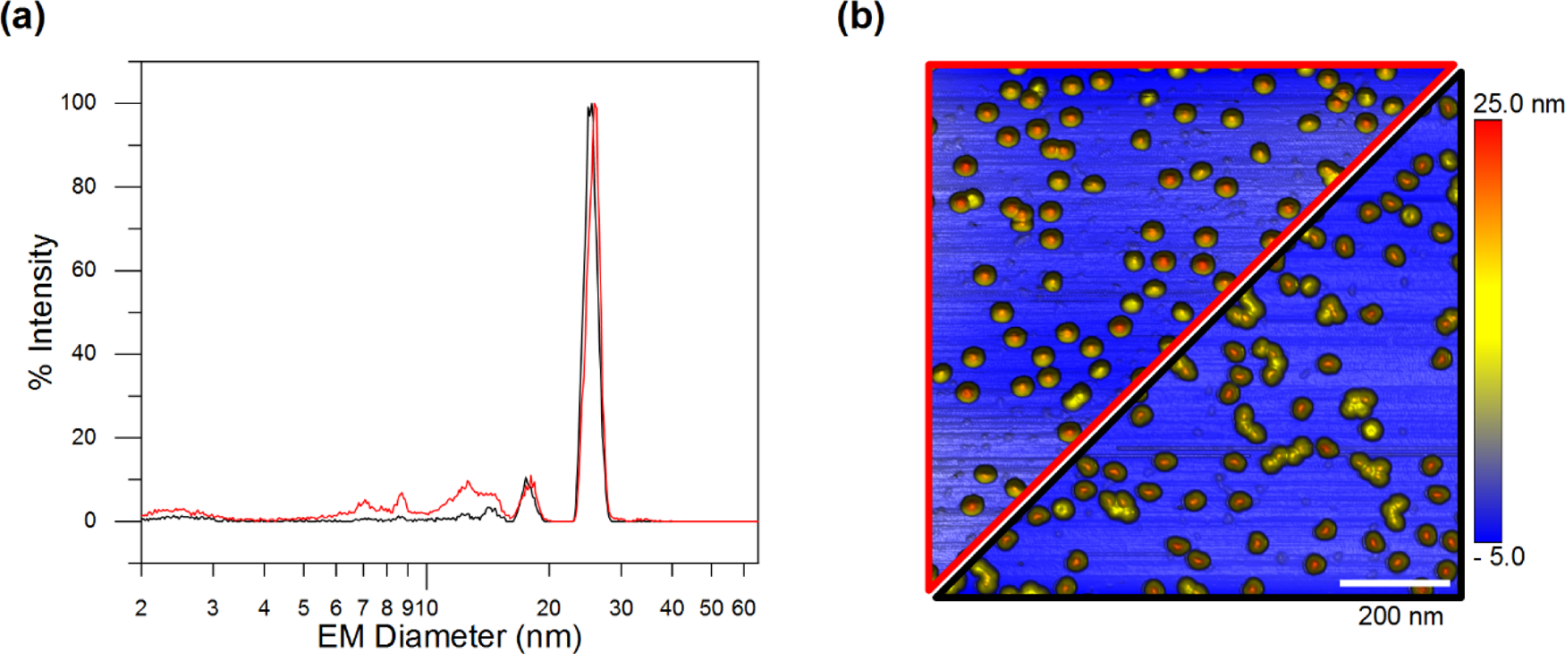
AAV8 VLPs carrying or lacking genomic information. (a) nES GEMMA spectra of empty (black trace) and filled (red trace) AAV8 VLPs. (b) AFM images of empty (right lower black triangle) and filled (left upper red triangle) AAV8 VLPs.

Subsequently, we expanded our nES GEMMA method for empty AAV8 VLPs to AAV8 VLPs encapsulating genomically engineered ssDNA (filled AAV8 VLPs). Figure 3a shows nES GEMMA of both empty and filled AAV8 VLPs after buffer exchange. Filled capsids (red profile) show a slight but evident shift toward higher EM diameter values of its peak’s apex when compared to the empty capsids (black profile). At the same time, the peak width (i.e., at full width half-maximum) of both bionanoparticles remains constant at approximately 2.2 nm. Ideally, since the proteinaceous structure of the capsid is identical as well as the diameter size, both preparations should yield the same EM diameter regardless of the presence or absence of the cargo material.

Differences in surface drying during the nES GEMMA electrospray/charge reduction process at atmospheric pressure might account for the EM diameter difference between empty and filled AAV8 VLPs. Especially for biological samples, the lack of the water solvation layer might destabilize capsids’ proteins by leading to a slight shrinking effect of nanoparticles and thus a decrease in EM diameter. The absence or presence of encapsulated ssDNA might enhance or minimize this shrinkage effect accordingly, leading to the observed difference between the two nanobioparticle types. In addition, the genome occupancy in the capsid will likely act as a supporting agent, thus providing counter-acting force against shrinking. In detail, nES GEMMA measurements with a statistical population of over 5000 detected capsids report EM diameters of 25.10 ± 0.18 nm for empty AAV8 VLPs and 25.93 ± 0.07 nm for the filled ones (see Table 1).

**Table 1:** nES GEMMA and AFM Statistical Analysis of AAV8 VLPs

	empty	filled
nES GEMMA		
total VLP count	5189	5611
average EM diameter (nm)	25.10 ± 0.18	25.93 ± 0.07
AFM		
total VLP count	557	525
average diameter (nm)	30.7 ± 2.4	25.8 ± 2.4
average height (nm)	22.6 ± 1.7	23.2 ± 1.3

Based on the assumption that the ssDNA acts as a scaffold agent for filled capsids, we also investigated via AFM instrumentation if noticeable differences were present between empty and filled AAV8 VLPs. Due to the dry environment conditions during AFM measurements, deformation effects (e.g., shrinking and deflation) should likely happen in the same fashion as we observed in nES GEMMA, especially for empty capsids. The results of this analysis are depicted in Figure 3b (Figure 3b is composed of two separate experiments). On top (i.e., red border triangle), filled AAV8 VLPs are shown, meanwhile empty ones are depicted on the bottom display (i.e., black border triangle).

As reported in Table 1, the statistical analysis on more than 500 capsids via AFM reports that the average diameter for empty capsids is 30.7 ± 2.4 nm, while that for the filled ones is 25.8 ± 2.4 nm. Moreover, average heights are 22.6 ± 1.7 and 23.2 ± 1.3 nm for empty and filled capsids, respectively. Considering that the only difference between the two preparations is the presence (or absence) of a genomic cargo, this further supports the assumption that the encapsulated genome in filled AAV8 VLPs has the effect of making the capsids more firm and less prone to size changes or shape distortions caused by different interactions with the mica surface or by the AFM tip. Empty capsids, in contrast, appear to be flexible and capable of forming numerous interactions with the mica surface, causing the sphere-like shape of the VLP to flatten and deform into an ellipsoid shape.^22^ In addition, the flattening value *f* measures the compression of a circle, or a sphere, along a diameter to form an ellipse, or an oblate spheroid, respectively (*f* = 0 for a circle or sphere). This property is determined by the following expression:
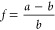
1where *a* is the larger dimension (e.g., semimajor axis) and *b* is the smaller dimension (e.g., semiminor axis). By using the values reported in Table 1, where *a* is the average diameter and *b* is the average height, we can calculate that for the filled capsids *f*_filled_ = 0.11, while for the empty ones *f*_empty_ = 0.36. Hence, filled VLPs are more spherical than empty VLPs. Therefore, the encapsulated genome is likely to act as an obstacle against capsids’ distortion, causing the filled capsids to retain a closer sphere-like structure than their empty counterparts, which is reflected in nES GEMMA data and AFM results alike.

## AF4 Fractionation Followed by Subsequent nES GEMMA and AFM Analysis

**Figure 4:**
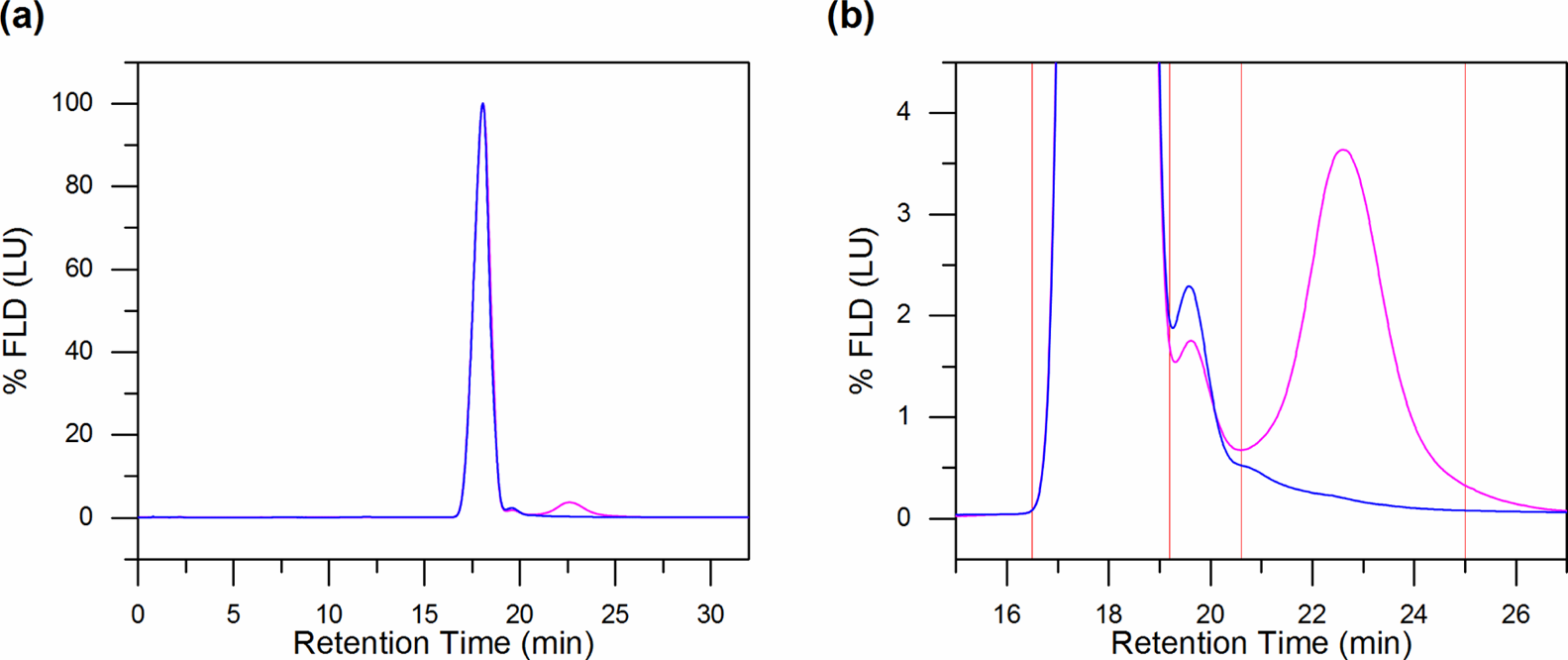
Stressing procedure and fractionation of the sample. (a) AF4 fractogram of control (blue trace) and heat/mechanical-stressed (magenta trace) sample. (b) The red vertical lines mark the collected fractions: 16.5–19.2 min monomer fraction; 19.2–20.6 min dimer and trimer fraction; 20.6–25.0 min higher oligomeric fraction.

The last aim of this work was to show the strategy outlined in Figure 1b, performing a cross-platform analysis of empty AAV8 VLPs over AF4, nES GEMMA, and AFM, especially under conditions of thermal/mechanical stress exerted on the bionanoparticles. Figure 4a shows the fractogram obtained by AF4, where purified samples, either stressed (i.e., magenta trace) or unstressed (i.e., blue trace), were analyzed. Samples’ stress conditions were achieved by means of agitation along with temperature alteration according to the Experimental Section. A magnification of Figure 4a is shown in Figure 4b. Here, it is easier to distinguish the fractogram peaks. The blue trace is composed of two peaks, a dominant one, which has a signal generated by the VLPs’ monomer, and a second smaller peak. Although its oligomeric state is not completely resolved, as it will be shown later in this work, this peak is characterized by a high abundance of VLPs’ dimers. Last, the magenta trace, besides showing comparable peak shapes as the blue trace, shows an extra peak at a retention time of 23 min. This peak, as it will be shown later, is composed of a heterogeneous mixture of VLPs in a higher oligomeric state. From now on, we will refer to these three peaks as monomer, dimer, and oligomer fractions, accordingly. Moreover, in Figure 4b, the vertical lines mark the time windows for the collection of the respective fractions. Hence, between retention times of 16.5–19.2 min, the monomer fraction was collected; from 19.2 to 20.6 min, the dimer fraction was collected; and last, the oligomer fraction was collected from retention times 20.6–25 min.

After fraction collection, aliquots of each fraction were subsequently analyzed by means of AF4 in order to verify the correct separation and their quality (see Figures S2–S4 in the Supporting Information).

**Figure 5:**
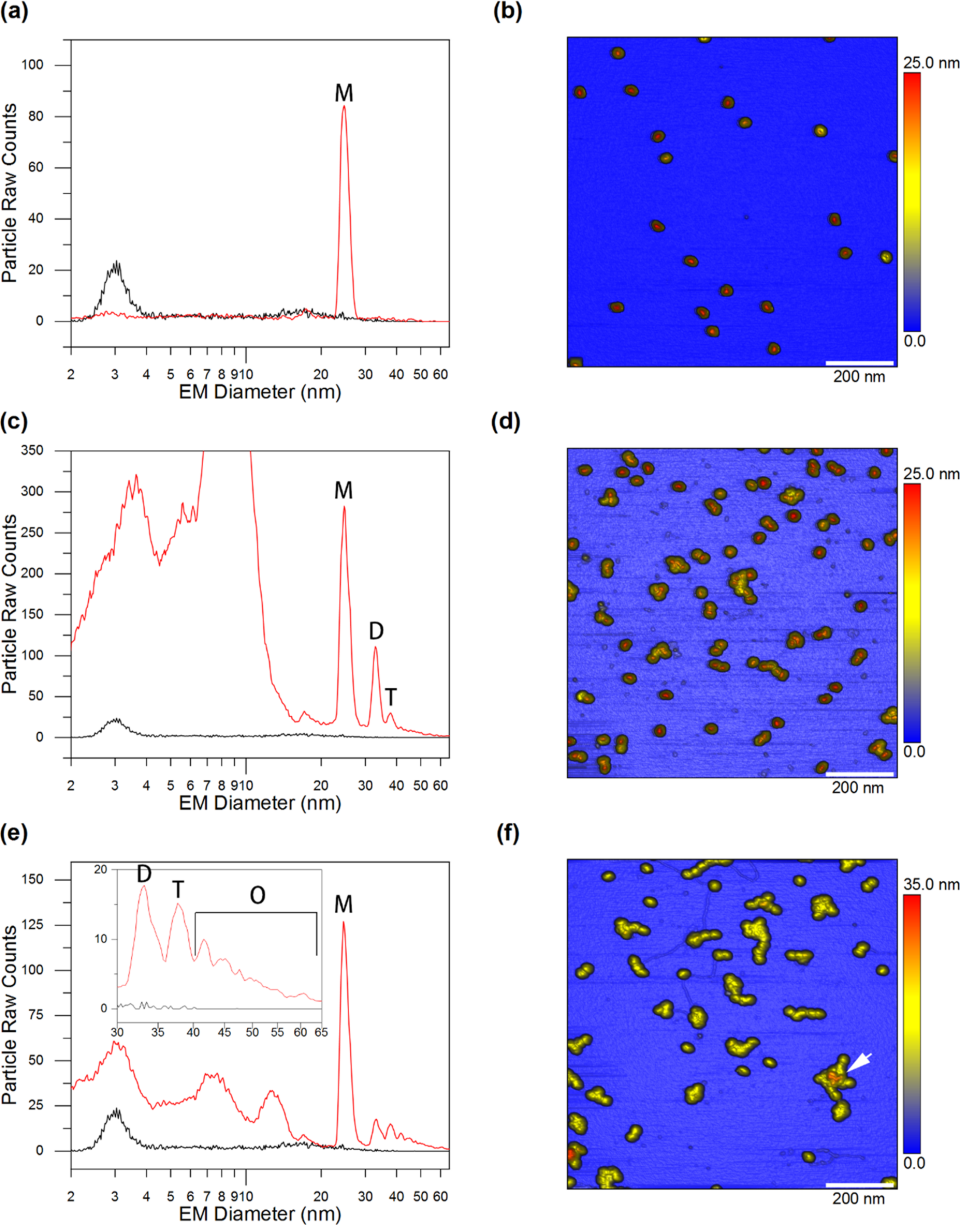
nES GEMMA spectra and AFM images of the three fractions collected with the AF4 technique (see Figure 4b). (a, b) Monomer (M) fraction; (c, d) dimer (D) and trimer (T) fraction; and (e, f) higher oligomeric (O) fraction. nES GEMMA spectra compare signals obtained for VLP-containing samples (red traces) and blanks recorded for a NH_4_OAc blank (black traces), respectively. nES GEMMA signals below 20 nm EM diameter putatively correspond to incompletely removed, aggregating AF4 buffer components. In (f), the arrow indicates an AAV8 VLP higher oligomer with a height of 35 nm.

The last step, as shown in Figure 1b, is the analysis of the collected fraction via nES GEMMA and AFM. The results are presented in Figure 5. For both instruments, the operating conditions are reported in the Experimental Section.

For the monomer fraction (retention time 16.5–19.2 min), Figure 5,ba presents nES GEMMA and AFM analyses on the monomer fraction of empty AAV8 VLPs, respectively. The nES GEMMA spectrum shows, as expected, a dominant peak with an EM diameter of 24.82 nm (i.e., label m), which matches the results reported in Table 1 and Figure 3a for both peak’s shape and apex center. In addition, from the AFM measurements, we can observe the presence of solely single nanoparticles, which corroborates our expectation.

For the following fraction (retention time 19.2–20.6 min), the nES GEMMA spectrum, as shown in Figure 5c, reports the presence of two further peaks after the monomer peak. These peaks are reported at EM diameters of 33.03 nm (i.e., label d) and 37.69 nm (i.e., label t), and they likely represent dimeric and trimeric AAV8 VLP nanoobjects, respectively. This interpretation is further validated by the AFM analysis reported in Figure 5d. Here, both monomers and dimers are nicely represented; trimers, as expected, are present to a lower degree, and few unresolved oligomers can also be found. The latter species, in particular, are likely to be responsible for the tailing signal after 40 nm EM diameter in the nES GEMMA spectrum (see Figure 5c). The presence of such species is due to the partial overlap of the oligomer fraction with the dimer fraction during AF4 separation.

For the final fraction (retention time 20.6–25 min), the analysis of this fraction by nES GEMMA (see Figure 5e) shows the signal associated with the residues of monomers, dimers, and trimers (i.e., labels m, d, and t) as dominating peaks in the spectrum. A fourth peak, with an apex center at 41.77 nm, might be associated with a tetrameric nanoobject; this and further oligomeric nanoobjects are sure to be present (i.e., label o in the inset of Figure 5e). However, due to the high heterogeneity of the sample and low abundance, these peaks become too broad and too low to be resolved under the selected device conditions. Moreover, based on the AFM structures visible in Figure 5f of this fraction, it is corroborated that plenty of these oligomeric nanoobjects are either simply out of the employed nES GEMMA instrument sizing settings (e.g., white arrow in Figure 5f, average section diameter 104 nm) or not abundant enough to generate a detectable peak.

Interestingly, in all of the three fractions analyzed by nES GEMMA, the monomer peak is ubiquitous; this could be explained by the fact that some capsids might have formed only weak interaction within the oligomeric nanoobjects; hence aggregation is reversible and the VLPs return in solution as single entities after AF4-based collection or during sample storage.

## Concluding Remarks

In this work, we presented a cross-platform analysis of purified AAV8 VLPs, which have been analyzed by three orthogonal techniques, namely, nES GEMMA, AF4, and AFM. Especially for nES GEMMA, removal of nonvolatile buffer components via buffer exchange is a critical step to ensure accurate measurements and to improve signal reliability. In this respect, nES GEMMA proved to be a valuable and suitable technique for AAV8 VLP characterization, and it can provide consistent and reproducible sizing data.

The AF4 technique, although in this case not able to fully resolve the oligomeric nanoobjects derived from the heat/mechanic-stressed AAV8 VLP empty preparation, was relatively easy to implement. Especially, its ability to fractionate the three different detected peaks has to be highlighted; the collected fractions were later successfully analyzed by means of nES GEMMA and AFM. Both techniques were able to detect monomeric, dimeric, and oligomeric AAV8 VLP nanoobjects. Moreover, AFM images confirm nES GEMMA findings and also show further larger oligomeric nanoobjects that were not analyzable with nES GEMMA due to the selected conditions focused on analyte resolution.

Moreover, nES GEMMA statistical evaluation over the EM diameter for both empty and filled AAV8 VLPs reveals the impact of the genomic material packed inside the capsid on the overall diameter of the particle. This characteristic is even more noticeable in the statistical analysis obtained by the applied AFM technique. Here, the lack of genomic material in the empty VLPs produces an even higher deformation of the capsids and thus corroborates nES GEMMA findings.

AF4, AFM, and nES GEMMA all proved to be valuable methods for the characterization of VLP and for gathering information in terms of surface-dry bionanoparticle size, sample purity, and VLP aggregation. These results are important to further expand our knowledge about the behavior of these particles during analytical investigations.

## Experimental Section

### Chemicals, Electrolyte Solutions, and Buffers

Ammonium acetate (NH_4_OAc, ≥99.99%) and ammonium hydroxide (ACS reagent) were both purchased from Sigma-Aldrich (Steinheim, Germany). The GEMMA electrolyte solution was prepared by dissolving 40 mM ammonium acetate with water of ultrahigh quality (UHQ) delivered by a Simplicity UV apparatus (18.2 MΩ × cm at 25 °C, Millipore, Billerica, MA, USA). The solution was adjusted to pH 7.0 with ammonium hydroxide and filtered through a surfactant-free cellulose acetate membrane with 0.20 μm pore size syringe filters (Sartorius, Göttingen, Germany).

AF4 carrier buffer (PBS) was prepared by dissolving sodium chloride (≥99.5%), monopotassium phosphate (≥99.0%), potassium chloride (≥99.5%, all from Sigma-Aldrich), and disodium phosphate (≥99.5%, Merck, Darmstadt, Germany) in UHQ water. The elution buffer additionally included 0.02% (w/v) sodium azide (Merck) as an antimicrobial agent. The pH was adjusted to 7.4 with ammonium hydroxide and filtered through a 0.1 μm pore size polyethersulfone membrane filter (VacuCap, Pall, NY, USA).

Sample preparation for AFM measurements required UHQ water and nitrogen gas (≥99.999%, Messer Austria GmbH, Gumpoldskirchen Austria) for rinsing and drying.

### Samples

Purified AAV8 VLP samples were provided by Baxalta Innovations (Orth/Donau, Austria, part of Takeda). Two different batches were provided: (i) so-called empty AAV8 VLPs (3776 μg/mL, i.e., 7.3 × 10^14^ capsids/mL), with 93% of capsids not carrying any genomic information, and (ii) so-called filled AAV8 VLPs (85 μg/mL, i.e., 1.6 × 10^13^ capsids/mL), where 66% of all the capsids were carrying a genomic load. The percentage of capsid filling was assessed via cryo transmission electron microscopy (CryoTEM).

### Stressing Conditions

The purified empty and filled AAV8 VLPs preparations, either after the buffer exchange step or directly from the stock, were subjected to a temperature stress of 65 °C and to mechanical shear conditions (i.e., 850 rpm agitation) by means of a thermomixer device (Model 22331, Eppendorf, Hamburg, Germany) for a fixed time of 10 min.

### Instrumentation

Nanoelectrospray gas-phase mobility molecular analyses were carried out on a TSI Inc instrument (Shoreview, MN, USA), which consisted of a nanoelectrospray charge reduction source unit (model 3480) including a ^210^Po charge equilibration device, an electrostatic classifier control unit equipped with a nanodifferential mass analyzer (nano-DMA; model 3080), and an *n*-butanol-driven ultrafine condensation particle counter (CPC; model 3025A) for AAV8 VLP detection. For the spraying process, the nES unit is equipped with a 24 cm long polyimide coated fused-silica capillary with an inner diameter of 25 μm (Molex, Lincolnshire, IL, USA). The capillary is manually cut and tapered with a homebuilt grinding machine based on the work of Tycova et al.^15^

AF4 experiments were performed on an Agilent 1200 system (Agilent Technologies, Santa Clara, CA, USA, auto sampler, pump, and detector), which consisted of an auto sampler, HPLC pumps, an AF4 separation device (Wyatt Technology, Santa Barbara, CA, USA), and a fluorescence detector (λ_ex/em_ = 280/340 nm). Wyatt Eclipse 3+ A4F (Wyatt Technology, Santa Barbara, CA, USA) was coupled to the system to control the AF4 channel, which was equipped with a 30 kDa molecular weight cutoff cellulose membrane (Superon, Wyatt Technology, Santa Barbara, CA, USA).

AFM experiments of the samples were imaged with a NanoScope III Multimode SPM instrument (Veeco Instruments, Santa Barbara, CA, USA) using silicon cantilevers with integrated silicon tips (NanoWorld, Neuchâtel, Switzerland, Arrow type: NC).

### nES GEMMA Operating Conditions

For nanoparticle separation and detection, the filtered air flow on the nES generator was set to 1.6 × 10^–5^ m^3^/s (1 liter per minute, Lpm), the CO_2_ gas flow to 1.6 × 10^–6^ m^3^/s (0.1 Lpm, 99.5% from Messer, Gumpoldskirchen, Austria), and the differential capillary pressure at 27.58 kPa (4 pounds per square inch differential). Capillary conditioning was performed by pre-spraying each sample for at least 3 min before starting the measurement. Capillary rinsing was performed by infusing the electrolyte solution until no signal from the previous sample was detectable. The sample was infused at a flow rate of 70 nL/min. The voltage at the capillary tip was set in order to have a stable Taylor cone (approximately 2 kV voltage and −380 nA current). The electrostatic classifier was set in automatic scanning mode (up scan time 120 s, retrace time 30 s) with a sheath gas flow rate of 2.5 × 10^–4^ m^3^/s (15 Lpm), which yielded a standard range of measurable electrophoretic mobility (EM) diameters between 1.95 and 64.9 nm. A total of 10 scans for each sample were used to generate a median spectrum. Mathematical and statistical calculations on the nES GEMMA spectra were made with the software OriginPro 9.1 (OriginLab, Northampton, MA, USA).

### nES GEMMA Sample Preparation

Buffer exchange against 40 mM NH_4_OAc for nES GEMMA was carried out by means of 10 kDa MWCO centrifugal filters (polyethersulfone membrane from VWR, Vienna, Austria). After three repetitions of spin filtration at 9000*g* each, the estimated final concentration for empty AAV8 VLPs was 22 μg/mL, while for filled AAV8 VLPs, it was 8.5 μg/mL.

### AF4 Operating Conditions

The AF4 method employed consists of three main steps: sample injection, sample focusing, and elution. The sample was injected for 2 mins at a constant flow rate of 0.2 mL min^–1^; this flow was kept constant also during the focusing step. The flow for the focusing step was set to 3.0 mL min^–1^. The focusing step was prolonged after the sample injection for an additional minute in order to reduce the lateral distribution of the sample itself. Last, the elution was performed with a crossflow decreasing in a linear fashion from 6 to 1 mL min^–1^ during 16 min of analysis. At all times, a baseline crossflow (i.e., detector flow) of 1 mL min^–1^ was present in the AF4 channel.

### AF4 Sample Preparation and Fraction Collection

Due to the limited sample concentration and availability of filled AAV8 preparation, AF4 analyses were carried out only with empty AAV8 VLPs. Empty capsids were either simply diluted from the stock solution or treated with the same procedure required for nES GEMMA. From the diluted stock solution, the concentration used was 377 μg/mL, while, after the buffer exchange step, the estimated concentration was 189 μg/mL (calculated via a calibration curve obtained from stock dilutions, data not shown).

For the aggregation experiment, aliquots from the stock solution were stressed as previously described. Following AF4 separation, a total of seven fractions from each peak-specific oligomeric state were manually collected: 24.5 mL for the monomer fraction, 8.4 mL for the dimer and trimer fraction, and 37.1 mL for the oligomeric fraction. The fractions were accumulated with 10 kDa MWCO centrifugal filters (cellulose membrane, Amicon Ultra-4, Merck Millipore, Darmstadt, Germany), at 9.0 × 10^3^ g ranging from 10 to 20 min. Once all collected fractions were accumulated into a single centrifugal filter, the buffer exchange against 40 mM NH_4_OAc was performed twice. As a final step, the same centrifugal filter was used to further concentrate the sample, by setting the centrifuge at 9.0 × 10^3^ g and by periodically checking until the volume of the retentate was at the 100 μL mark of the filter unit.

### AFM Operating Conditions

The images were acquired in tapping, constant amplitude mode at a scanning rate of 1.99 Hz, over a scan area of 1 μm^2^.

### AFM Sample Preparation

The freshly split mica platelet was first tested by AFM to verify the smoothness and homogeneity of its surface. The sample deposition method involves spotting 10–20 μL of the sample (5–20 μg/mL solutions) on the platelet’s surface at room temperature; to allow adsorption of the analytes, the sample is left resting for 5 min undisturbed before being gently rinsed with UHQ water and successively dried under a soft stream of nitrogen gas (outlet pressure 3.5 bar). Last, the mica platelet is reinserted on the AFM’s piezoelectric scanner and it is ready for analysis.

### AFM Image Analysis

The AFM images have been analyzed by NanoScope Analysis 1.5 software (Bruker, Santa Barbara, CA, USA) by applying the same approach used elsewhere for AFM characterization of AAV VLPs.^36^ In particular, the monomeric AAV8 particles’ height and diameter have been characterized by selecting all the particles above a defined height (i.e., half of the maximal height of the entire single particle population) and by excluding boundary nanoparticles, small ones (i.e., under 18 nm of diameter), and aggregates. A demonstration of particle selection is available in Figure S5 in the Supporting Information.

## Supporting Information Available

Additional AF4 fractograms acquired to confirm the quality of the collected fractions and example of particle selection for AFM image analysis (PDF)
