# Supplementary material for: Adeno-associated Virus Virus-like Particle Characterization via Orthogonal Methods: Nanoelectrospray Differential Mobility Analysis, Asymmetric Flow Field-Flow Fractionation, and Atomic Force Microscopy
Source: ACS Omega. 2021 Jun 15;6(25):16428–37. doi: 10.1021/acsomega.1c01443 (PMC8246466; doi:10.1021/acsomega.1c01443)
Supplement: Supplementary file 1 — ao1c01443_si_001.pdf [file ao1c01443_si_001.pdf]

Supplementary Material

Adeno associated virus-like particle characterization via orthogonal methods: nES-DMA, AF4, and AFM

Samuele Zoratto[1], Victor U. Weiss[1], Gernot Friedbacher[1], Carsten Buengener[2], Robert Pletzenauer[2], Alexandra Foettinger-Vacha[2#], Michael Graninger[2], Guenter Allmaier[1,*]

[1] Institute of Chemical Technologies and Analytics, TU Wien (Vienna University of Technology), Vienna, Austria

[2] Pharmaceutical Sciences, Baxalta Innovations (part of Takeda), Vienna, Austria

[#] Current affiliation: Analytical Development, Böhringer Ingelheim, Vienna, Austria

Keywords: Gene therapy platform, AAV 8, VLP, nES GEMMA, DMA, AF4, AFM.

Correspondence (*): Guenter Allmaier, Institute of Chemical Technologies and Analytics, TU Wien, Getreidemarkt 9/164, A-1060 Vienna, Austria

E-mail: guenter.allmaier@tuwien.ac.at

Tel: +43 1 58801 15160

Fax: +43 1 58801 16199

The supplementary material includes:

(i) an additional AF4 fractogram (relative plot) demonstrating that the sample composition in terms of AAV8 VLP monomers and oligomers remains constant upon buffer exchange.

(ii) fractograms of AAV8 VLP monomer, dimer, trimer and higher oligomer fractions.

(iii) an example of particle selection for AFM image analysis.

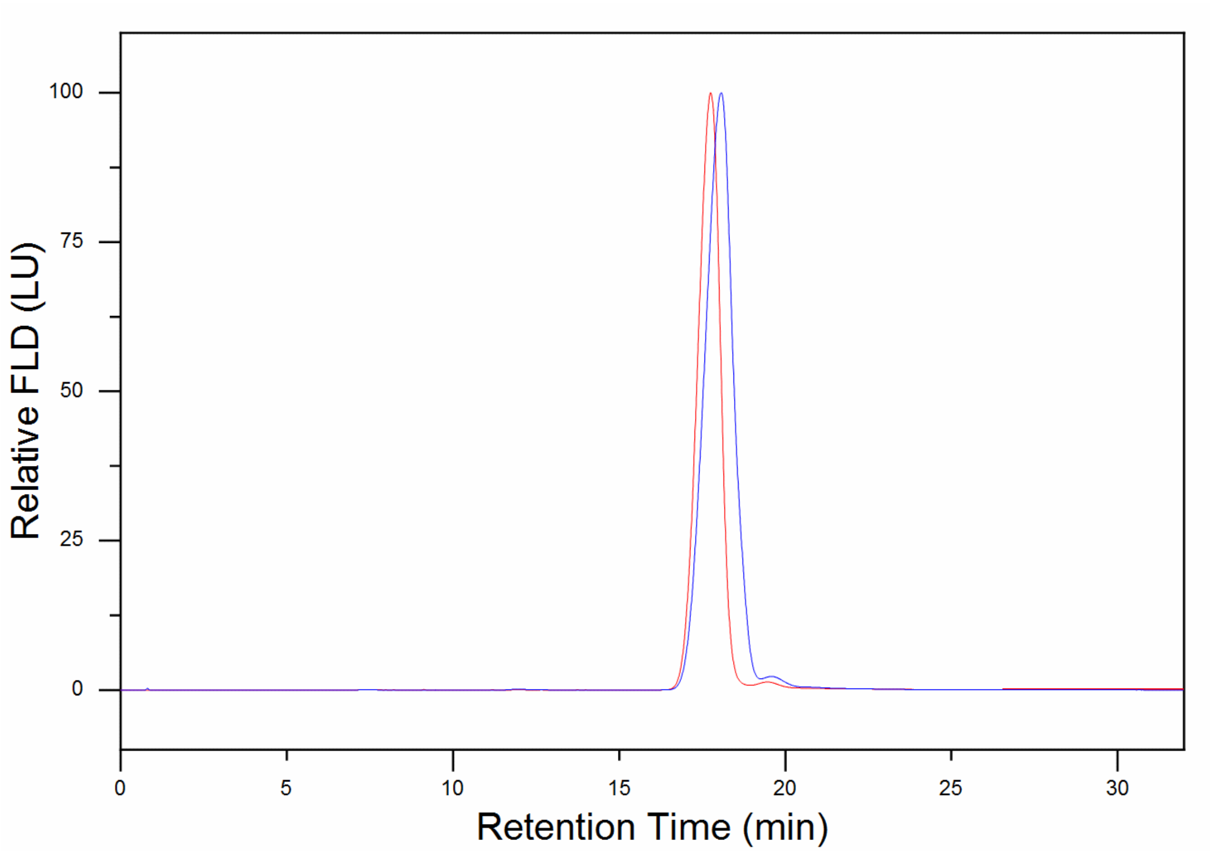

Figure S1. Fractogram overlay of empty AAV8 VLPs either from simple dilution (blue trace) or after buffer exchange step (red trace).

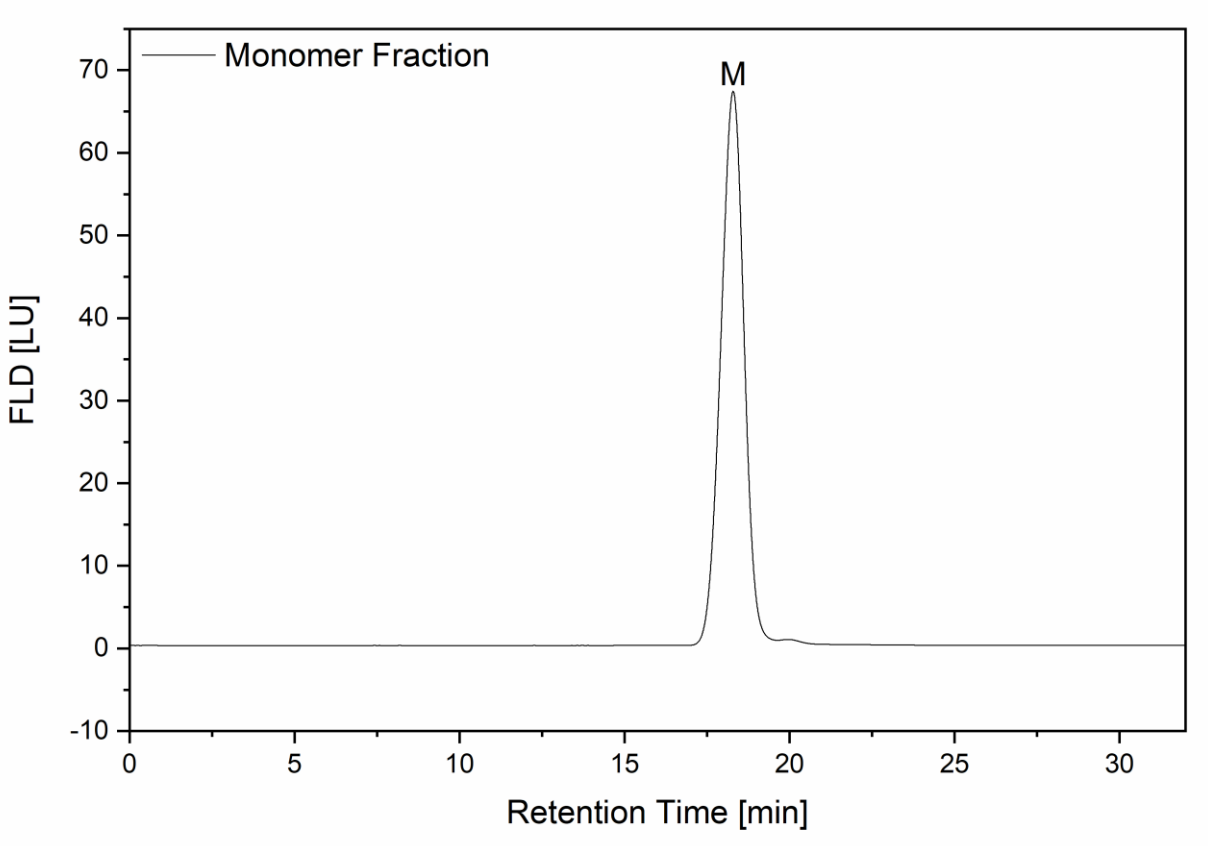

Figure S2. Fractogram of the monomer fraction (empty AAV8 VLPs) after collection and concentration steps (M, monomer).

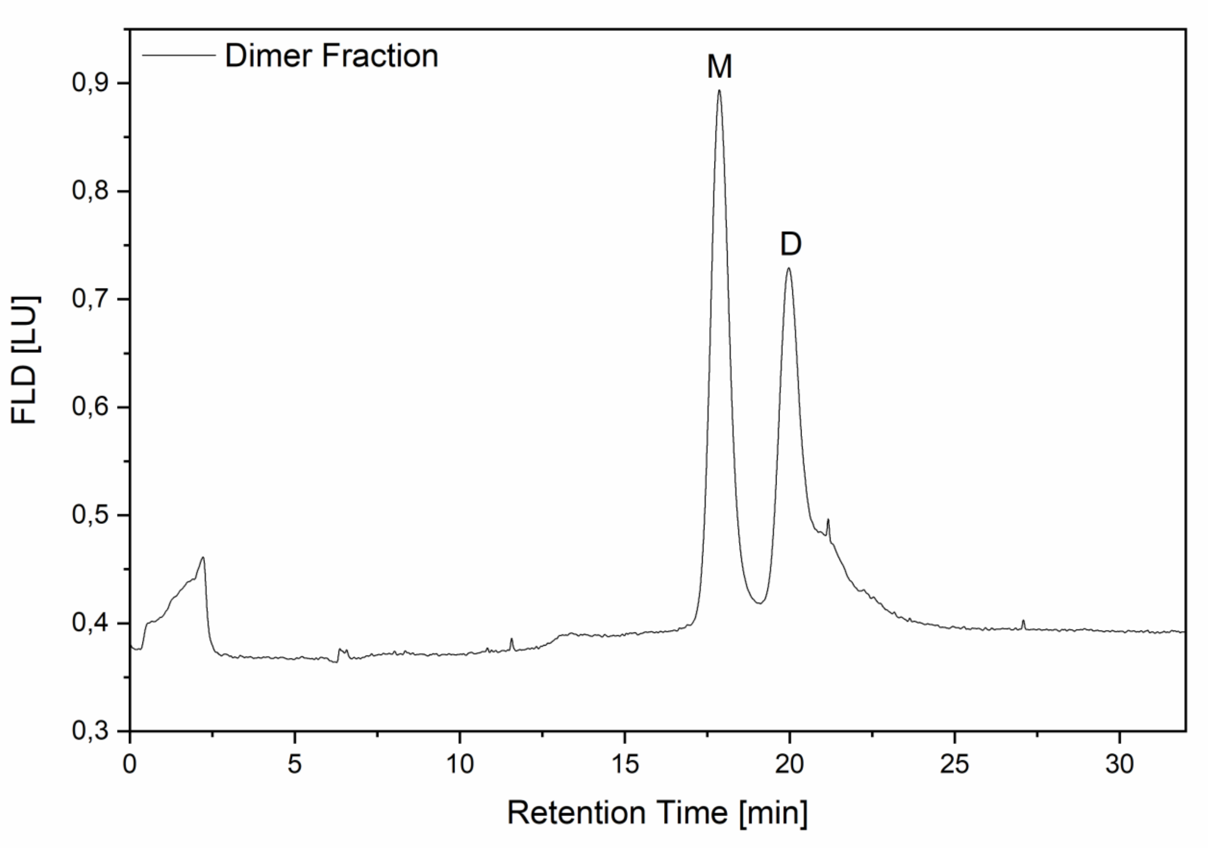

Figure S3. Fractogram of the dimer/trimer fraction (empty AAV8 VLPs) after collection and concentration steps (M, monomer, D, dimer).

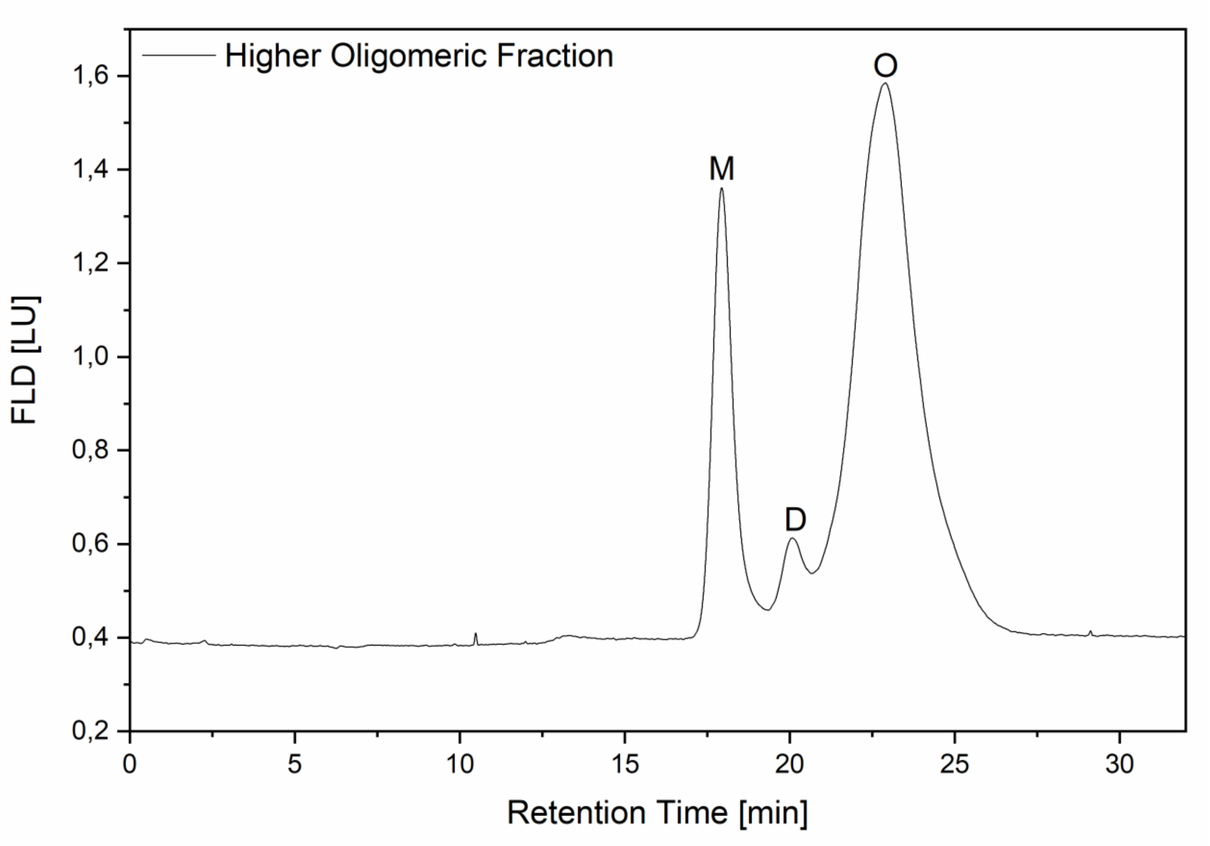

Figure S4. Fractogram of the higher oligomeric fraction (empty AAV8 VLPs) after collection and concentration steps (M, monomer, D, dimer, O, oligomer).

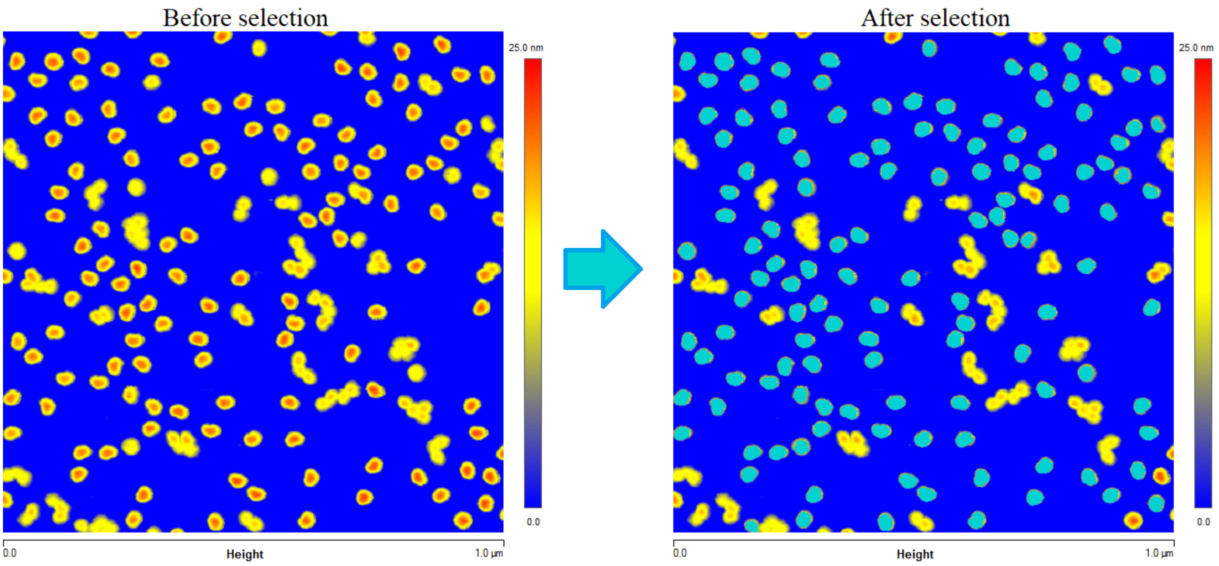

Figure S5. Example of particle selection with NanoScope Analysis 1.5 software. Only particles meeting the criteria described in the materials and methods section (colored in light blue) are used for the statistical analysis.
